# Quantitative evaluation and reversion analysis of the attractor landscapes of an intracellular regulatory network for colorectal cancer

**DOI:** 10.1186/s12918-017-0424-2

**Published:** 2017-04-05

**Authors:** Yunseong Kim, Sea Choi, Dongkwan Shin, Kwang-Hyun Cho

**Affiliations:** Department of Bio and Brain Engineering, Korea Advanced Institute of Science and Technology (KAIST), Daejeon, 34141 Republic of Korea

**Keywords:** Cancer reversion, Logical model, Systemic approach, Colorectal cancer, Reversion target, Attractor landscape evaluation, Functional network motif, Systems biology

## Abstract

**Background:**

Cancer reversion, converting the phenotypes of a cancer cell into those of a normal cell, has been sporadically observed throughout history. However, no systematic analysis has been attempted so far.

**Results:**

To investigate this from a systems biological perspective, we have constructed a logical network model of colorectal tumorigenesis by integrating key regulatory molecules and their interactions from previous experimental data. We identified molecular targets that can reverse cancerous cellular states to a normal state by systematically perturbing each molecular activity in the network and evaluating the resulting changes of the attractor landscape with respect to uncontrolled proliferation, EMT, and stemness. Intriguingly, many of the identified targets were well in accord with previous studies. We further revealed that the identified targets constitute stable network motifs that contribute to enhancing the robustness of attractors in cancerous cellular states against diverse regulatory signals.

**Conclusions:**

The proposed framework for systems analysis is applicable to the study of tumorigenesis and reversion of other types of cancer.

**Electronic supplementary material:**

The online version of this article (doi:10.1186/s12918-017-0424-2) contains supplementary material, which is available to authorized users.

## Background

Cancer is one of leading causes of death according to the World Health Organization since 2000 [1]. The most common features of cancer are uncontrolled proliferation, epithelial-mesenchymal transition (EMT), and stemness [2]. Although these features and their underlying mechanisms were extensively studied, cancer still remains as a complex disease with no single cure probably due to a lack of systematic approach. Cancer is evolved from normal cells which are transformed by a number of genetic mutations accumulated in various signaling pathways during tumorigenesis [3]. Such alterations often make cancer cells more robust than normal cells to an external apoptotic stimulus such as targeted drugs in therapeutic approach. Apoptosis is the programmed cell death, and triggering it is the ultimate therapeutic goal for most cancer. However, tumor-selective apoptosis is not always possible, and it is more likely to cause side effects to normal cells. Therefore, there have been increasing demands for an alternative anti-cancer therapeutic strategy by which the uncontrolled state of cancer cells could be reversed back to the normal cellular state while preventing normal cells from undergoing apoptosis [4]. Finding this strategy based on cancer reversion requires the development of a systemic analysis for quantitative evaluation of a cancerous state. Through such an analysis, we could comprehensively characterize important features of cancer and also identify promising drug targets for cancer reversion.

Since the first observation of cancer reversion phenomenon from teratoma into normal cells in 1907 [5], numerous reports have shown that cancer reversion can occur both in vitro and in vivo [6–8]. For instance, inhibition of overexpressed PPARγ has been shown to trigger differentiation of colorectal cancer which is the second leading cause of cancer mortality in the United States [9]. However, previous studies were limited to present the phenomenon itself without providing a systematic or mechanistic analysis. To reveal the underlying mechanism of cancer reversion and to discover specific targets for the reversion in a systematic way, a simplified but essential molecular regulatory network model needs to be constructed [10, 11].

The behavior of a cellular system is caused by complicated interactions of molecules that form a complex molecular interaction network. The steady states of all the molecules can be represented by an attractor state of the network which corresponds to a cellular phenotype [12]. However, indeed, the functional properties and phenotypes of a cell are mainly determined by the steady states of specific important molecules, not all the molecules [13]. Cancer can be understood as a functional disease triggered by the disturbance of such steady states of functional molecules by the accumulation of somatic mutations [14]. The complexity of the molecular interaction network implicates the existence of inherent functional redundancy. So, there might exist some control targets that can drive the cancerous cellular states into normal-like states although multiple molecules in the network are already disturbed by the occurrence of mutations during tumorigenesis [15]. In this respect, cancer reversion might be realizable by converting the steady state of important molecules to that of a normal cell through a systems approach [16].

In this study, we have constructed a simplified but essential molecular regulatory network model of a colorectal cancer cell to investigate the underlying mechanism of cancer reversion. Most previous studies focused on specific mutations accumulated in various signaling pathways which were frequently observed with cancer reversion phenomena in colorectal cancer [17, 18]. We have manually integrated such information through an extensive survey of literatures and databases, and identified essential regulatory interactions between key molecules. We have employed a Boolean network model to describe the logical relationship of molecular interactions. Then, we have explored the cancer reversion mechanism by systematically perturbing all of the molecular components in our network model and evaluating the resulting changes of the attractor landscape with respect to uncontrolled proliferation, EMT, and stemness. From this analysis, we have identified a set of promising control targets for cancer reversion, and some of them are in accord with previous experimental observations. Moreover, we have analyzed functional network motifs that are relevant to the target nodes and their stability in order to understand the underlying mechanism of cancer reversion. As a result, we found that cancer reversion can occur by the alleviating the robustness of the intracellular gene regulatory network. Therefore, our study provides new insight into the cancer reversion mechanism through a systematic way of identifying the control targets.

## Methods

### Construction of an essential molecular regulatory network model of colorectal cancer

**Fig. 1:**
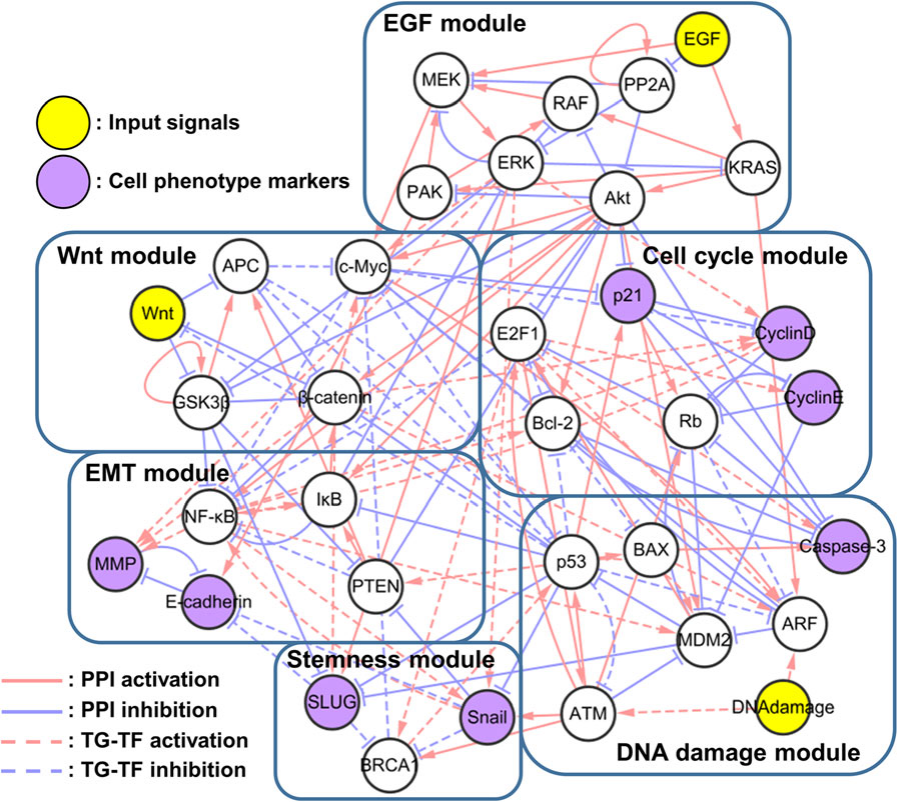
The network model with 34 nodes and 135 edges to explore colorectal cancer reversion phenomena. Each node represents the biological molecules such as proteins, mRNAs or transcription factors while each link represents either activation (*red*) or inhibition (*blue*). A dashed-link represents an interaction between transcription factors and target genes while a solid-link indicates a protein-protein interaction. Three input signals are colored in *yellow*, and eight cell state markers are in *purple*. The network can be subdivided into six functional modules in round squares

To investigate the mechanism of cancer reversion in a system-level approach, we have constructed a simple but essential molecular regulatory network (34 nodes and 135 links) of colorectal cancer from Kyoto Encyclopedia of Genes and Genome (KEGG) database, by integrating major signaling pathways in colorectal cancer (Fig. 1). Based on these signaling pathways, we have added three input nodes (EGF, Wnt and DNA damage) that are closely related to colorectal cancer. In addition, eight of marker nodes (Snail, SLUG, MMP, E-cadherin, CyclinE, CyclinD, p21 and Caspase-3) also have been included in order to classify biological states of resulting attractors in the network. These marker nodes have been shown to be highly associated with uncontrolled proliferation, EMT, and stemness [19–21]. We also added additional direct or indirect links based on literature surveys and protein-protein-interaction (PPI) data inferred from Genome-scale Integrated Analysis of Gene Networks in Tissues (GIANT) [22] so that our network model was confirmed to fit well with the input-output relationship from the previous studies. Moreover, the entities of matrix and vectors in weighted sum logic are tuned with action mechanisms through literature surveys. All the literatures referred for constructing the network are summarized in Additional file 1. The detailed descriptions of constructing the regulatory network model of a colorectal cancer cell are summarized in Supporting Text. S1 of Additional file 2.

### Network update logic formation based on biological action mechanisms

After constructing the network, we have implemented the updated logics of our Boolean network by converting the Boolean operator’s equation based on biological action mechanisms into weighted sum logics (Additional file 3 and Supporting Text. S2). In weighted sum logic, a network topology with N-nodes can be defined according to an N-by-N connectivity matrix, ***M***_***C***_, where each matrix element (***M***_***C***_)_*ij*_ represents the connection from node j to node i. The tendency of each node’s state without any incoming signal can be represented as a value in the basal level of a column vector, ***V***_***B***_ ⋅ Mutation profiles and copy number alterations (CNAs) for individual patients obtained from the big bang model of Sottoriva et al., the Human Protein Atlas (HPA) and The Cancer Genome Atlas (TCGA) databases, were projected by adjusting the entity of ***V***_***B***_ ⋅ For each discrete time point t, the state of each node i, denoted ***V***_***i***_^***t***^, can be either 0 or 1. The input to each node is given by the weighted sum vector, ***W***_***s***_ = ***M***_***C***_ ⋅ ***V*** ^***t***^ + ***V***_***B***_ ⋅ The next state ***V***_***i***_^***t*** + 1^ is then determined by the weighted sum of inputs to node i, (***W***_***s***_)_***i***_, as follows:

$$ {\boldsymbol{V}}_{\boldsymbol{i}}^{\boldsymbol{t}+1}=\left\{\begin{array}{c}\hfill 1\hfill \\ {}\hfill {\boldsymbol{V}}_{\boldsymbol{i}}^{\boldsymbol{t}}\hfill \\ {}\hfill 0\hfill \end{array}\right.\kern0.5em \begin{array}{c}\hfill {\left({\boldsymbol{W}}_{\boldsymbol{s}}\right)}_{\boldsymbol{i}}>0\hfill \\ {}\hfill {\left({\boldsymbol{W}}_{\boldsymbol{s}}\right)}_{\boldsymbol{i}}=\mathbf{0}\hfill \\ {}\hfill {\left({\boldsymbol{W}}_{\boldsymbol{s}}\right)}_{\boldsymbol{i}}<0\hfill \end{array} $$

The connectivity matrix in which basal levels of the weighted sum logics and mutation profiles for simulation are summarized in Additional file 4. The nodes are synchronously updated at each time step. We also attempted to apply the asynchronous update rule and found that the resulting point attractors and their basins were conserved in both update rules. Since the overall basin size of point attractors has reached more than 80% of all the initial states in our result, we concluded that the phenotypic features of attractor landscapes from both update rules were similar. The biological and analytical validities of synchronous update logic are explained in Additional file 2: Figures S1. The entities of the connectivity matrix are translated from the logical equations of the Boolean operators with our custom code using Matlab® 2014b, while the entities of basal column vector are driven from experimental data. The source codes for mathematical simulations are included in Additional file 5.

### General concepts of simulation analysis on the network model

For large-scale networks, it is usually difficult to obtain the whole state transition diagram by a full search because of the computational limitations. Therefore, in our network model (*N* = 34), we estimated the attractor landscape by investigating the state transition diagram from a number of randomly sampled initial states such as 100,000. This initial population was sufficiently large for reliable reproduction of major attractor states, and thus the phenotypic scores of attractor landscapes were varied within very small (±2%) deviation from the average score of 100 repetitions. During the perturbation analysis and validation of our network model, node perturbations and mutation profiles were implemented by pinning the state of the corresponding nodes into either 0 or 1. To qualitatively evaluate the various input-output relationships of our network, we have employed the proposed method from Helikar et al. [23]. In this simulation, the activation ratio of eight marker nodes was measured as increasing the stochastic activity levels of three input nodes independently.

### Quantitative normal-like score evaluations of attractor landscapes

**Fig. 2:**
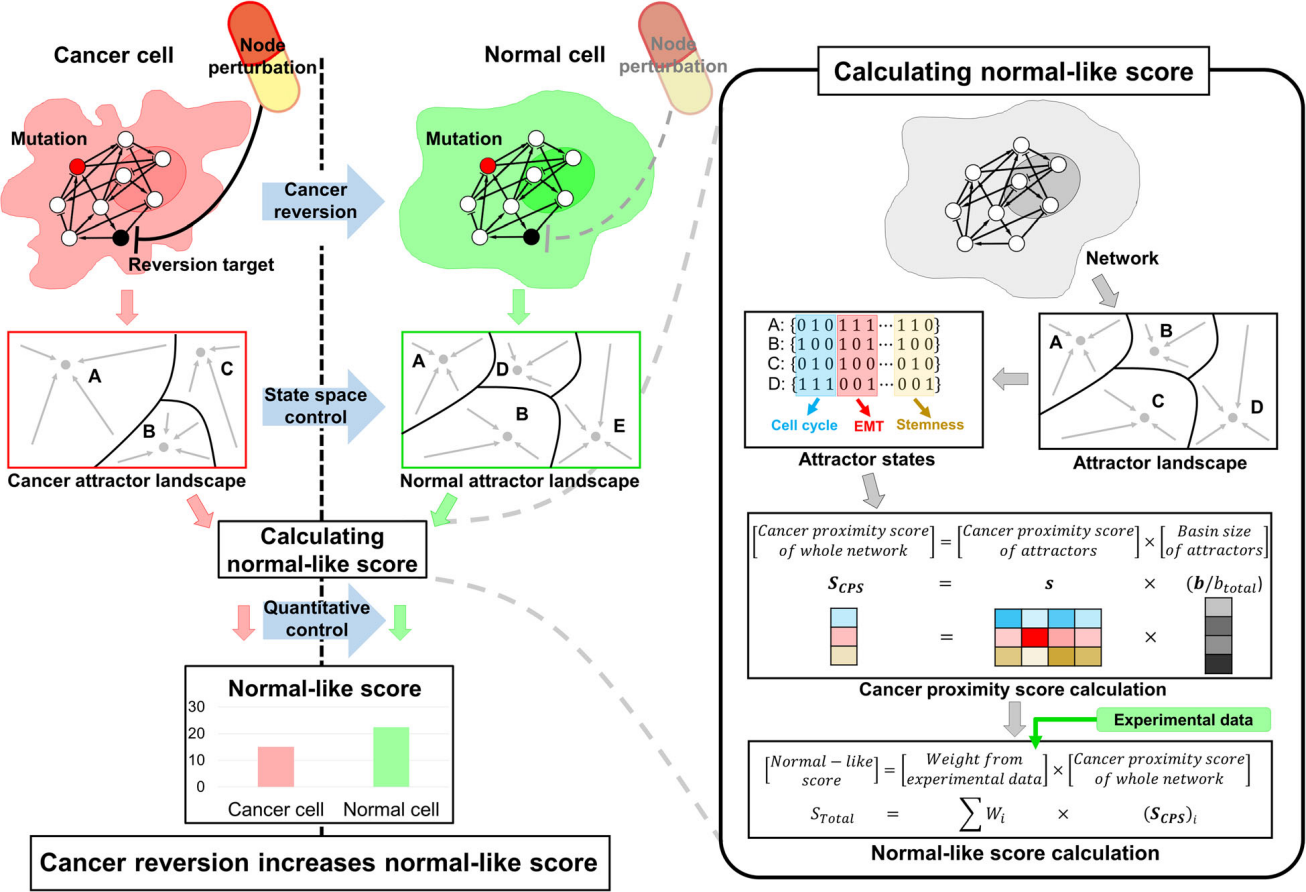
A workflow of quantitative evaluation of attractor landscape. To reveal the effective cancer reversion target, we calculated a normal-like score for a given *attractor landscape*. The normal-like score represents the quantitative similarity of an attractor landscape with that of normal cells in regard to three phenotypic aspects of cancer such as proliferation, EMT, and stemness. Every attractor was scored by adding up the scores of these three aspects and multiplied by the ratio of basin size of attractors among the entire initial states with the given weights from experimental data. The probable cancer reversion targets can be identified by systematically perturbing each node in the network and selecting the perturbed nodes that increase the normal-like score after the perturbation

**Fig. 3:**
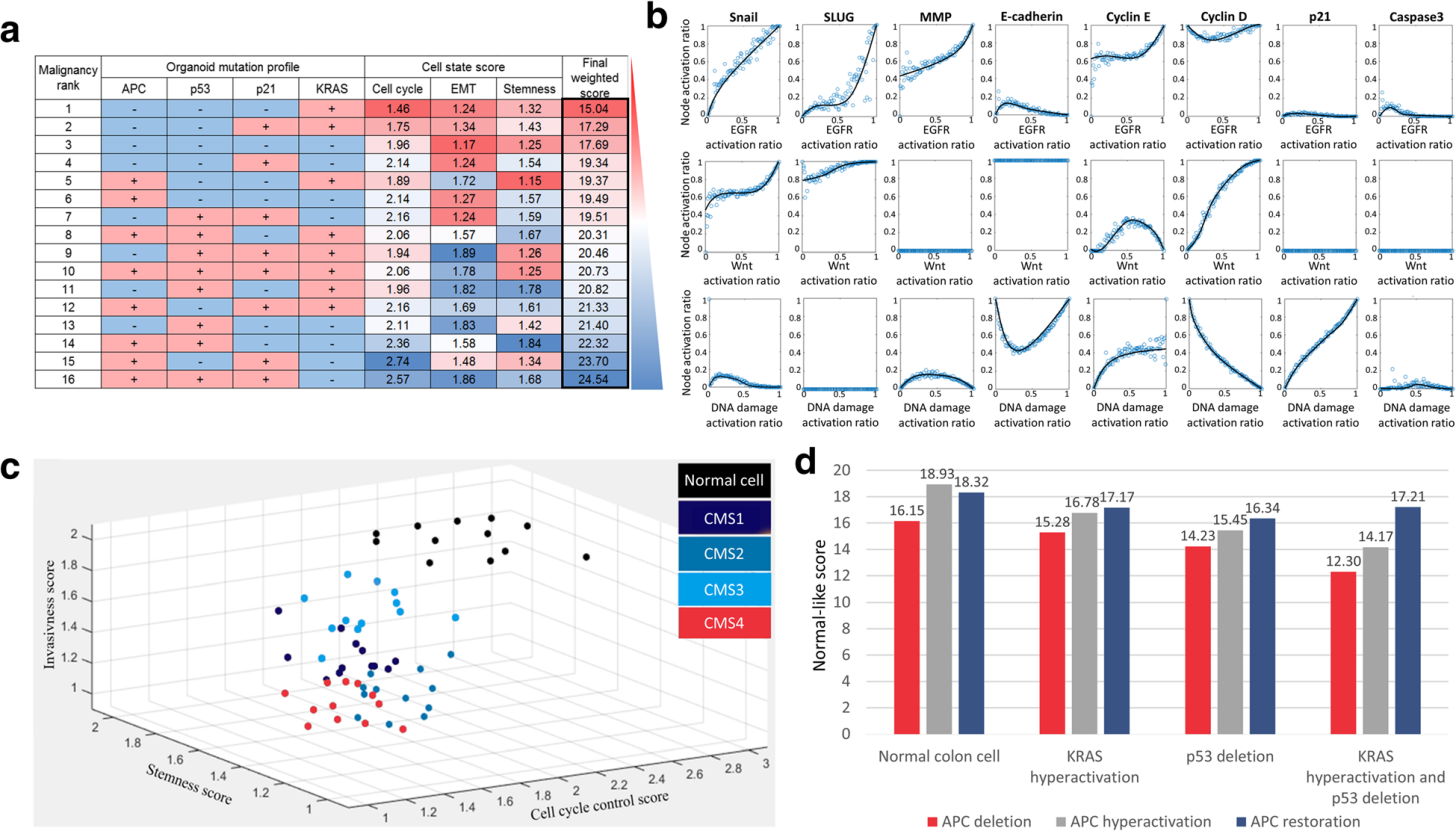
The validation of the proposed network through the demonstration of cancer reversion phenomena. **a** Data-based weight fitting results from the scoring system. We have ranked the order of malignancy for each mutation profile based on the experimental data (*first column on the left*). The three cell state scores for each aspect were obtained from the attractor landscape simulations after projecting each mutation profile to the network (*middle three columns*). To fit the cell state score ranks with the experimental data, each cancer proximity score is weighted and summed. As a result, the colorectal cancer network with the same mutational profile from the experimental data was sorted in similar ranks with the experimental results (*last column on the right*). **b** Various qualitative input-output relationships obtained from the proposed network model. The activation ratio of eight marker nodes has been measured along with the increase of the three input nodes independently. As a result, the input-output response curves are well in accord with the previous experimental data. **c** Clustering the CMSs in the space of cancer proximity scores according to the mutation profile of each subtype. 12 normal colon cell networks with various expression fold changes (*black dots*) are well clustered in the upper region of the three-dimensional space in our scoring system. After the projection of CMS mutation profiles in colored dots, the network models were well clustered according to the CMS in the three-dimensional space of cancer proximity scores. Moreover, the CMS4 network models (red dots) were predicted to be in the most malignant state, which agrees with the previous studies [29]. **d** Cancer reversion phenomena mediated by APC restoration. The reversion phenomenon was observed in terms of the normal-like score recovery to that of a normal colon cell after the APC restoration of colon cancer cell. The increase of normal-like score due to the APC restoration (difference of normal-likes scores in last red and blue column on the right) is much higher than those of APC hyper-activation (*difference of normal-likes scores in last red and gray column on the right*), KRAS restoration (*difference of normal-likes scores in third and last red column from the left*) and p53 restoration (*difference of normal-likes scores in second and last red column from the left*)

To quantify the degree of malignancy of cancer, we have introduced a scoring system for the attractor landscape in our network based on the combined state of eight marker nodes. The scoring system allowed us (i) to match each of the resulting attractors with functional proximity to the cancer cell phenotype, (ii) to quantify the malignancy score of cancerous states, and (iii) to quantitatively calculate a single “normal-like score” of the all attractor landscape. An attractor landscape of network is characterized by attractor states and their corresponding basins of attraction. By using our scoring system, each attractor state can be quantified based on three “cancer proximity scores (CPSs)” (proliferation, EMT, and stemness) which are determined by the combined activity of eight marker nodes of the corresponding attractor state. Low scoring means “proximate to cancerous”, whereas high scoring indicates “normal-like”. We calculated a vector of CPSs for an attractor landscape (***S***_***cps***_ = ***s*** ⋅ ***b***/*b*_*total*_), where ***s*** represents a matrix composed of every individual CPSs of each attractor and ***b***/*b*_*total*_ represents a vector of the relative basin size of each attractor. The detailed workflow of the scoring process for an attractor landscape was shown in Fig. 2. To obtain the normal-like score of a cancerous state based on the normal-like scoring system, we have used CRISPR/Cas9 experimental data from previous studies in intestinal stem cells [24]. Jarno et al. compared niche factors dependency, crypt formation and invasiveness of organoids with CRISPR/Cas9-mediated modification of KRAS, APC, p53 and p21(SMAD4) genes. Thus, we were able to rank malignancy scores of each mutation profile by fitting the weights of the vector of CPSs (***S***_***CPS***_) with real data. As a result, the higher the value of the normal-like score, the higher the probability that the network attractors are analogous to their biologically normal state in reality. Thus, we determined the CPSs of network states in regard to three types of the hallmark of cancer, and quantitatively presented the normal-like score by summing CPSs multiplied by the given weights from Jarno et al. To simplify the weights into integers, we have set the weights of proliferation, EMT and stemness as six, four and one, respectively. The mutation profiles of organoids and their simulation analysis for normal-like scores are summarized in Fig. 3a.

### Node perturbation analysis to identify reversion targets

To identify the cancer reversion targets based on the normal-like score, we have performed a node perturbation analysis by systematically perturbing the molecular activity of each node in the network. There are three types of perturbation: activation, inhibition, and restoration perturbations. An activation or inhibition perturbation fixes the state of that node to 1 or 0, respectively, while a restoration perturbation restores the basal level of that node back to the value in normal colon cell. During a single node perturbation analysis, we have obtained several potent cancer reversion targets in the case of when the normal-like score of the network landscape has been increased after perturbation [25]. During a double nodes perturbation analysis, we have classified perturbation types into three: synergistic, additive and antagonistic. For instance, we measure how much the normal-like score increases after performing single- and double-node perturbations with node A and B. If the increase of normal-like score during the double-node perturbation is larger, similar or smaller than the sum of the normal-like scores from the individually perturbed nodes A and B, then each incident can be classified as synergistic, additive or antagonistic, respectively. The range between increased normal-like scores has been calculated as 20% in average after performing double node perturbation in various pairs. Additional explanations are in Additional file 2: Figures S2.

### Functional network motif analysis to explore the reversion mechanisms

Synergistic effects usually arise from emergent properties of a complex network due to the close interactions of molecules in the network such as feedback loops or network kernels [26]. Synergistic pairs of cancer reversion targets strongly interact with each other and constitute functional network motifs in which every node show a similar consequence in the normal-like score for the same node perturbation type. Moreover, fixing a node in the functional motif may decrease the ability of other nodes in the motif to increase the normal-like score by their perturbation. A functional network motif can be understood as a functional unit block for cancer reversion. If a node in a network motif is mutated, cancer reversion could not be achievable with any perturbations among other nodes in the motif. Rather, additional perturbation of nodes in the other network motifs could induce cancer reversion [27]. Based on these assumptions, we have extracted the functional network motifs by the procedure below. First, we calculated the increase of normal-like score by perturbing one of the cancer reversion targets, “A”. Second, we systematically fixed any single node and calculated again the increase of normal-like score by the node “A”. Third, we selected one fixed node, “B”, which mostly decreased the normal-like score, as a member of functional network motif with the node A. Next, we repeated this process with fixing the node B in order to find another node, “C”, which mostly decrease the normal-like score. Finally, we repeated these processes until the increased normal-like score by the perturbation of A became smaller enough to neglect. After determining the entire members of the functional network motif with this algorithm, we have identified the motifs from the original colorectal cancer regulatory network. After curating the network, we have tested the stability of each motif by comparing the averaged node activities over every attractor in colorectal cancer with those of perturbed colorectal cancer (see Additional file 2: Figures S3 for additional explanations about motif stability analysis).

### Robustness analysis of various sequences of mutations

To determine the robustness of the network against external perturbations, we examined the number of attractors and the average basin size of five major attractors. If the number of attractors is small or the average basin size of major attractors is large, external perturbation could hardly change the network state that stays in an attractor state, which makes the network robust to external perturbations [28]. During the robustness analysis, the entire attractor landscapes have been simulated while accumulating the node state fixation into either 0 or 1. We have investigated the robustness of a network against the external signals as mutations were accumulated, by tracking the changes in the number of attractors and average basin size of attractions from five major attractors (Additional file 2: Figures S4).

## Results

### Proposed network model recapitulating the various cancer reversion phenomena

With our logical network model of colorectal cancer, we have tested whether it can successfully recapitulate both the dynamics of colon cancer cell and various cancer reversion phenomena reported in previous studies [18]. First, using our logical network model, we have simulated the various input-output relationships observed in normal colon cells under various conditions. Figure 3b shows that the functional relationships between three input stimuli and eight cell state markers of the network in the previous studies are reproduced by our network model, confirming that our network model successfully implemented the dynamics of normal colon cell [19–21]. Next, we have projected the 12 mutation profiles sets randomly selected from four consensus molecular subtypes (CMS) of colorectal cancer into our network model to obtain 48 CMS network models [29]. Guinney et al. have clustered colorectal cancer into four distinctive subtypes based on the statistical differences of gene expression data. From evaluation of the CPSs for each CMS, the network models were well clustered according to the CMS in the three-dimensional space of CPSs (Fig. 3c). This result indicates that our network model was successfully transformed into that of a colon cancer cell with the mutation profiles. Thus, our three-dimensional scoring system is sufficient to capture phenotypic characteristics of colorectal cancer such as CMS profiles. Moreover, the CMS4 network models were predicted to be on the most malignant state, which is well in accord with the work of Guinney et al. [29]. To further test the validity of our network model, we have simulated the cancer reversion for colorectal cancer network models by restoring the basal level of APC. During the simulation, the mutation profiles were obtained from the patients’ data in Sottoriva et al. [30]. We observed that the reversion phenomenon was enhanced most significantly in cancer network with KRAS hyperactivation, APC deletion, and p53 deletion (Fig. 3d) [18]. Together, these results indicate that our network model and its dynamics effectively demonstrated the biological characteristics of normal colon cell and the cancer reversion phenomena in colorectal cancer.

### Systematic identification of cancer reversion targets by single node perturbation analysis

**Fig. 4:**
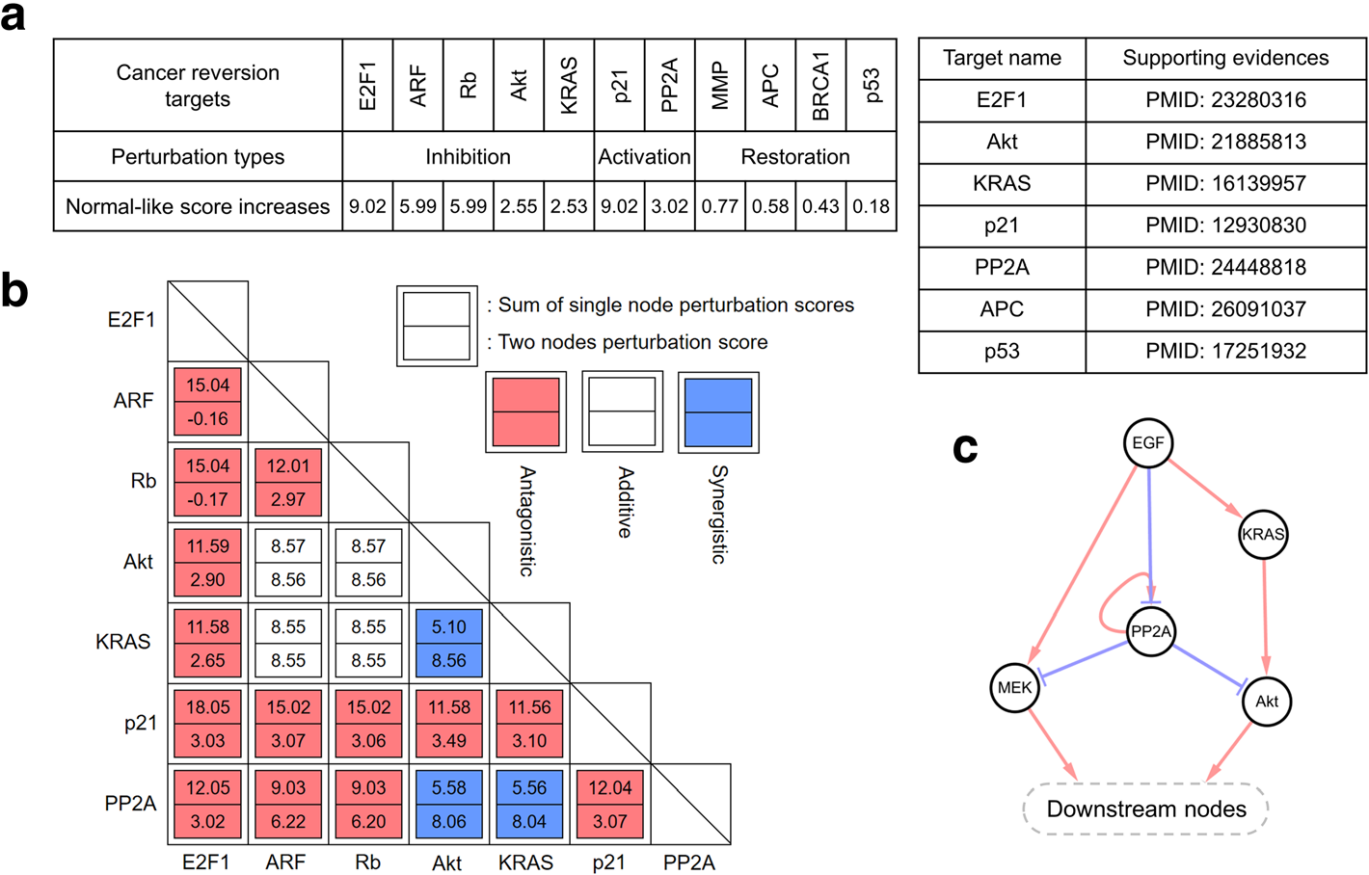
Probable cancer reversion targets identified from the single node perturbation. **a** Eleven probable cancer reversion targets with three types of perturbation, including activation, inhibition, and restoration, are listed (*left*). Seven of them are listed with the supporting evidence (*right*). **b** Double node perturbation simulation has revealed three synergistic pairs (*blue*) which were selected when the increase of normal-like score for two node perturbation is larger than the sum of each increase of normal-like score of single node perturbation. **c** The functional network motif related with synergistic pairs. The identified motif is a functional unit of EGF signal transduction which contains EGF and its downstream nodes

Next, we have performed the single node perturbation analysis to predict the cancer reversion targets (see the Methods section for detailed explanations of node perturbation analysis). The targets were selected such that the normal-like score of attractors increased for the three different perturbations of it, activation, inhibition, or restoration. Every node in the network has been perturbed individually and then compared with one another. As a result, we could identify a set of target nodes (E2F1, ARF, Rb, Akt, KRAS, p21, PP2A, MMP, APC, BRCA1, and p53), whose alterations can induce cancer reversion (Fig. 4a). These 11 suggested cancer reversion targets were further analyzed and verified to be robust against the structural or dynamical perturbations of the network (Additional file 2: Figures S5). Moreover, the statistical significance of these targets were analyzed to ensure the simulation results (Additional file 6 and Additional file 2: Figures S6). Among them, E2F1, Akt, KRAS, p21, PP2A, APC, and p53 have been reported to influence deeply cancer reversion in the previous studies (Fig. 4a). For instance, E2F1 is suggested to be a probable target of cancer reversion due to its function to decrease the recurrence of colorectal cancer and arrest the cell cycle [31]. The inhibition of Akt is also known not only to suppress colon cancer growth in vitro and in vivo but also to keep the homeostasis of colon tissue for its functional maintenance [32]. In addition, inhibiting KRAS mutation has shown to disrupt maintenance of cancer in mice [33]. Moreover, p21 has been suggested to control the cell cycle, and its activation eventually facilitated the recovery of the functional cell cycle control system in cancer cell [34]. APC restoration has stimulated cellular differentiation and reestablished crypt homeostasis in colorectal cancer [18]. Finally, restoring the loss of function of PP2A has previously shown to drive the phenotypic reversion of colorectal cancer [35]. Furthermore, our result suggests that other four targets (ARF, Rb, MMP, and BRCA1) could be potential targets for cancer reversion, even though they have not been previously elucidated (Fig. 4a). ARF and Rb, which are involved in controlling cell cycle, are known to decrease in expression levels in colorectal cancer and act as tumor suppressors [36]. MMP is an important marker in colorectal cancer since it not only regulates invasiveness but also affects prognosis in patients [37]. Moreover, the risk of colorectal cancer conferred by mutations in BRCA1 is recently increasing, implying that BRCA1 might play a key role in colorectal tumorigenesis [38]. Therefore, our results suggest that these four molecules could be potential targets that can restore cancerous states to normal state.

### Four stable network motifs that critically enhance the robustness of cancer cell networks

Our single node perturbation simulation has suggested 11 cancer reversion targets. We further analyzed double nodes perturbation to find the synergistic pairs among seven targets by applying inhibition or activation perturbation, where restoration perturbation was not considered due to the complexity of simulation analysis. As a result, we identified three synergistic pairs (KRAS-Akt, Akt-PP2A, and PP2A-KRAS) which were selected when an increase in the normal-like score for two node perturbation is larger than the sum of increases for individual node perturbations (Fig. 4b). The synergistic effects observed in double node perturbation simulation imply the existence of functional motifs that act as a functional unit block for cancer reversion. Thus, we have parsed the motif which is composed of the synergistic pairs among the cancer reversion target nodes. Interestingly, every node in synergistic pairs is included in the EGF signal transduction pathway. As a result, the synergistic pairs act together as a functional motif which controls the EGF signal propagation to the remaining network nodes ranked at the lower level of hierarchy (Fig. 4c). Our result indicates that perturbing any multiple nodes in the functional motif could induce synergistic effects on cancer reversion, which might be an effective strategy to trigger the cancer reversion with minimum number of control nodes. The double nodes perturbation results are summarized in Additional file 6 and Additional file 2: Figures S7.

**Fig. 5:**
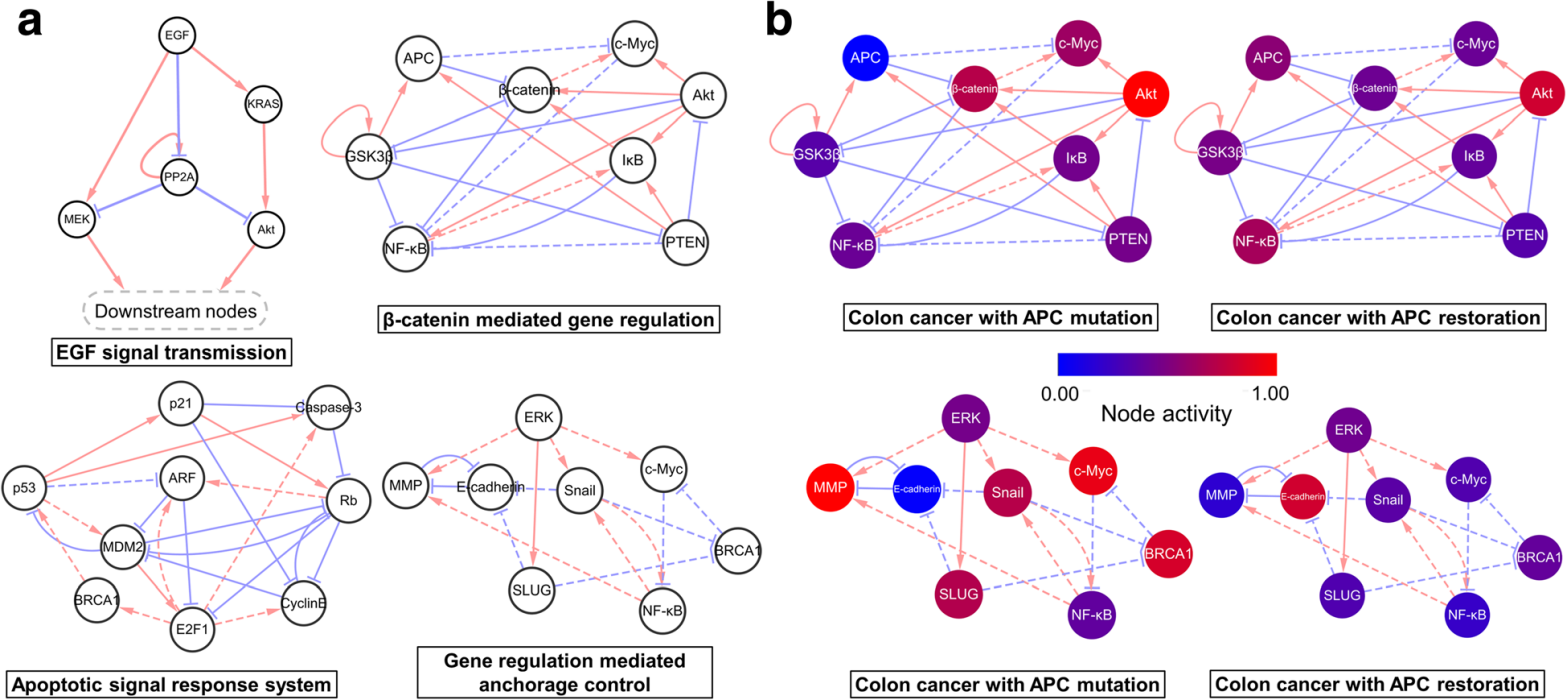
Four stable motifs of eleven cancer reversion targets. **a** All of the four motifs are relevant to the signal transduction pathways that are closely involved in colorectal cancer progression. **b** Changes of node activities for APC restoration in each motif. For APC mutation or restoration, the average expression level of some molecules is robustly fixed to 1 (*red*) or 0 (*blue*) regardless of external signals. However, the activities of some other nodes can fluctuate according to external signals, and thus show 0.5 (*purple*) on average

After the double nodes perturbation analysis regarding the mechanistic correlations between seven cancer reversion targets, we further analyzed functional motifs that contain these cancer reversion targets (see the Methods section for detailed explanations of functional motif analysis). The identified target nodes have constituted four functional motifs that are highly relevant to the gene regulatory pathways indicated in Fig. 5a. Fixing any node of the motifs to either 0 or 1 largely suppressed the increase of normal-like score caused by the perturbation of cancer reversion targets. In our study, the identified functional motifs are basic units of cancer reversion phenomena, which are closely related to cellular responses of cancer cells to external signals, such as growth signal response, EMT or cell cycle control signals. After grouping the predicted cancer reversion targets in four functional motifs, we have performed motif stability analysis to confirm the significance of four motifs. The basic principle for determining the stability of target nodes in each functional unit is to find a set of nodes that tend to converge on a specific state, 0 or 1, regardless of their initial states. The set of average node activities in four motifs have dramatically changed to the values of near 0.5 from 0 or 1 after perturbing cancer reversion targets (Fig. 5b and Additional file 2: Figures S3). If the functional motif in a cancerous state is robust to external signals, then the node activities of the motif would be insensitive to the external signals and thus converge to 0 or 1. In this regard, the node activities of near 0.5 after disturbing the cancer reversion targets means the decrease of the robustness to external perturbations. The perturbation of the predicted cancer reversion targets in our study has shown to decrease the stability of the functional motifs. Thus, these motifs became sensitive to the external signals, and easily changed the cell state and functions according to their environmental conditions. Together, our results indicate that cancer can be characterized as the stable functional motif that contributes to enhancing the robustness of cancer-related attractors, suggesting that inhibiting such a stability of functional motifs could be an efficient strategy for cancer reversion.

### The cancer reversion mechanism revealed by perturbing the stable network motifs

**Fig. 6:**
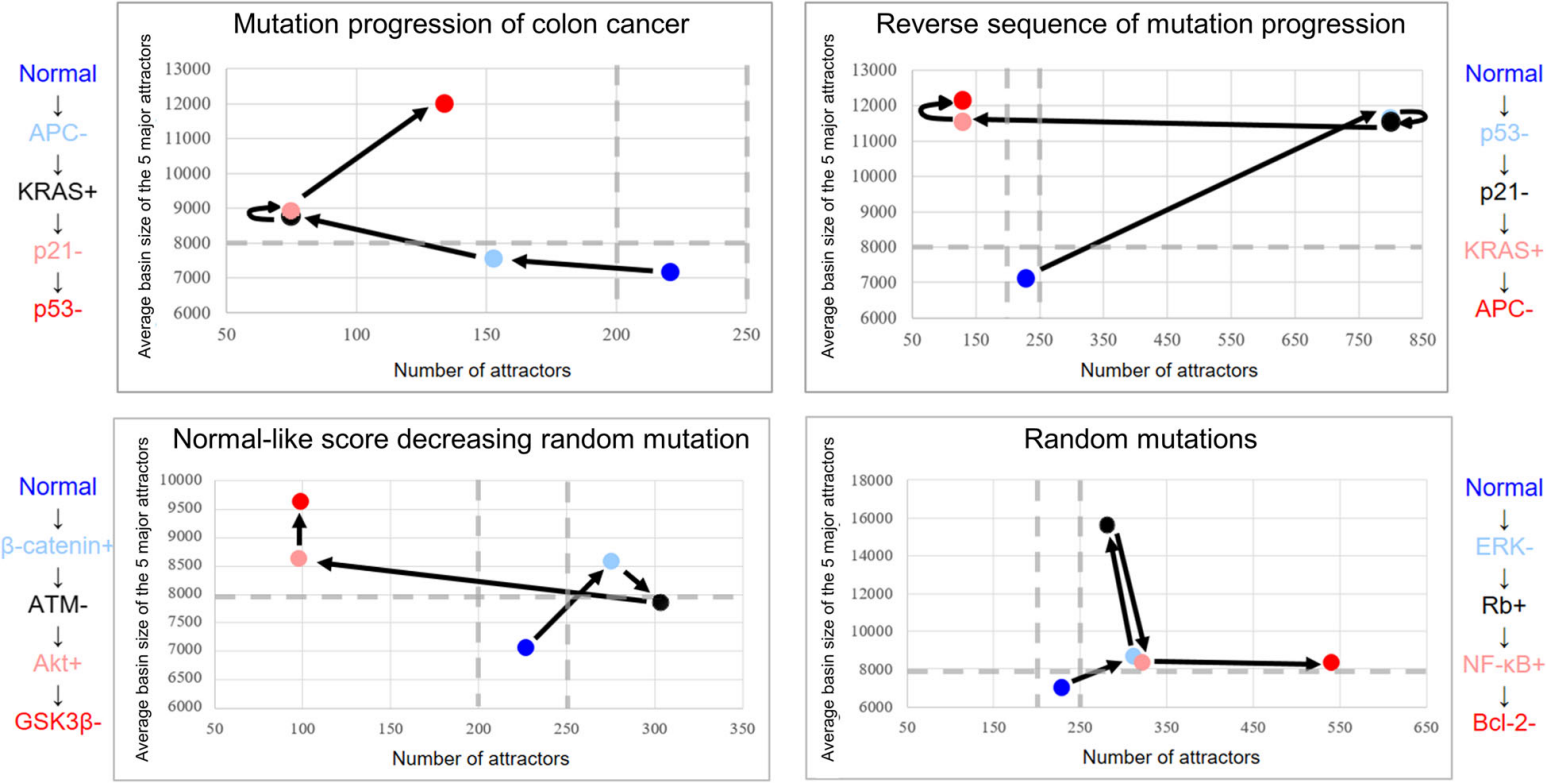
The robustness analysis of the sequential mutation accumulations. A network is in general expected to be robust against the external signal when the number of attractors decreases or the average basin size of the five major attractors increases. Thus, the network becomes robust when the dot moves toward the *left* or *upper* part of the coordinate plane. The results of the sequential accumulation of mutations in colorectal cancer tumorigenesis have revealed that the robustness of the network against the external signals gradually increases in each step of mutation accumulation (*top left*). However, reversing the sequence of mutations accumulated in colorectal cancer tumorigenesis caused the increase of the robustness near the last step of the sequence (*top right*). Moreover, when we considered randomly selected mutation sequences (*n* = 30) and determined each mutation type to decrease the normal-like score, it also caused the increase of the robustness near the last step of the sequences (*bottom left*). A representative trajectory of random mutation sequences is shown in the figure. Furthermore, most of the random mutation accumulation sequences have not shown any significant increase of the robustness along with the accumulations of mutations (*bottom right*)

Our motif stability analysis has revealed that the major difference between colon cancer cells and normal cells is the stability of functional motifs. Because the stability of functional motifs represents the responsiveness to the external signals, cancer cells have the characteristics of higher state stability than normal cells. This indicates that the cancer cells become robust against external perturbation during tumorigenesis, and thus controlling the robustness can reverse cancerous state to normal-like state. The network becomes more robust against external signals when the number of attractors decreases or a basin size of attraction increases. So, we had further analyzed the changes of robustness during the tumorigenesis by tracking the number of attractors and the average basin size of attraction in each step when mutations were sequentially accumulated (see the Methods section for detailed explanations of robustness analysis). The most commonly observed mutation sequence in colorectal cancer gradually increased the robustness of the network at each step of mutation accumulation compared to the case of randomly selected mutation sequences, whereas reversing such a sequence caused a transient increase of the robustness near the last step of the sequence (Fig. 6). Moreover, when we considered randomly selected mutation sequences and determined each mutation type to decrease the normal-like score, we also obtained a similar pattern with the reverse of the most observed mutation sequence in colorectal tumorigenesis. Furthermore, most of the randomly selected mutation sequences have not shown significant increases of the robustness as mutations were accumulated. These analyses indicate that the specific mutation sequences accumulated during colorectal tumorigenesis might be preferentially selected for a cancer cell to avoid sensitive responses to external signals, and therefore such a robustness would be a major characteristics of cancer cells. Thus, perturbation of the stable motifs by altering the reversion targets may decrease the robustness of cancer cells against the external signals. Taken together, our results suggest that the key strategy for cancer reversion is to increase the responsiveness of cancer cells to external signals by disturbing stable functional motifs of cancer cells.

## Discussion

The systemic view of cancer biology has been highlighted for a long time. The hallmarks of cancer have known to be associated with the complex dynamics of molecular interaction networks [39]. A variety of network models of cancer cells has been previously established to investigate such dynamic features of the network from a systems biological perspective. However, previous studies have mainly focused on qualitative assessments of cancer such as changes in input-output relationships in cancer cells or comparison of major attractors between normal and cancer cells [19, 40]. For instance, a recent study employed a network modeling approach to identify control targets for anti-cancer treatment by qualitatively examining the changes of basin size between major attractors, such as proliferation and apoptosis [40]. However, these system-based studies still have fundamental limitations in systematic control for cancer reversion because there is no quantitative evaluation of cancer status. In our study, the resulting normal-like score of a whole attractor landscape is determined by a set of several functional characteristics related to cancer malignancy and their weights that reflect the malignancy rank of experimental data. Thus, the normal-like score not only contains the phenotypic features of the whole attractor landscape but also quantitatively define the phenotypic malignancy. Moreover, the previous study based on the attractor ratio analysis has a limit in estimating whether a specific control will cause improvement or regression of malignancy due to a lack of quantitative evaluation of attractor landscape. Our scoring system is able to not only explore the most efficient drug target among multiple targets but also quantify its efficiency, which allows a systematic control for cancer reversion. Furthermore, the quantitative analysis of attractor landscapes based on the normal-like scoring system can be utilized for various types of cancer as well as colorectal cancer in order to identify effective control targets in a systemic approach. There still remains a limitation in our study in that the reconstructed essential regulatory network model is not complete although we have examined the robustness of our result (see Additional file 2). Hence, it would be a challenging future study to further expand the network model and validate it by experiments.

Dysfunctions of cancer cells arise from a series of mutations accumulated during tumorigenesis. The permanent alterations of a molecular interaction network by these accumulated mutations seem to prevent cancer reversion by locking cancer cells to the cancerous state. Although cancer reversion has been studied for decades and identified key molecules of high importance, it remains difficult to understand how cancer reversion can be achieved [4]. Current cancer therapies are usually performed in terms of tumor regression by inducing apoptosis in cancer cells. However, tumor regression is often accompanied by unwanted regression of normal cells, one of the frequent side effects of cancer treatments [41]. To overcome these side effects, we focused on restoring the functional phenotypes of cancer cells to those of normal cells rather than tumor regression. To achieve this, we have set the cell cycle arrest index to be of greater importance than that of apoptosis in order to approach cancer reversion without inducing apoptosis in normal cells. In addition to cell cycle arrest, the attractor landscape was assessed by EMT and stemness, representative functional phenotypes of cancerous states, to quantify the transformation to a normal-like state. Taken together, we suggest that the reverse control based on these phenotypes can return cancer cells to functionally normal cellular states, which can pave the way to new anti-cancer treatments.

## Conclusions

To investigate the underlying mechanism of colorectal cancer reversion from a systems perspective, we have constructed a logical network model of colorectal tumorigenesis by integrating key regulatory molecules and their interactions from an extensive survey of literature and experimental data. We established a Boolean network model and validated its logical rules for every node in order to create a biologically relevant molecular regulatory network that can mimic the signaling dynamics and tumorigenesis in a colorectal cell. By systematically perturbing each of the molecules in the network, we identified cancer reversion targets that can transform cancerous cellular states to normal-like states. Moreover, we quantitatively evaluated the resulting changes of the attractor landscape through our scoring system with respect to uncontrolled proliferation, EMT, and stemness. Interestingly, many of the identified molecular targets were well in agreement with previous studies. We further revealed that the identified targets constitute functionally stable network motifs that contribute to enhancing the robustness of attractors in a cancerous state against various external regulatory signals. Our study provides a new approach to understanding colorectal tumorigenesis and offers promising targets that can drive cancer reversion.

## Additional files

Additional file 1: The colorectal cancer regulatory network comprising 34 nodes and 135 links. (XLSX 42 kb)

Additional file 2: Supporting texts and figures. (PDF 908 kb)

Additional file 3: The Boolean logic operator equations and the conversion of logic operator into weighted sum logic. (XLSX 34 kb)

Additional file 4: The connectivity matrix of the weighted sum logic, basal levels of the nodes, and mutational profiles. (XLSX 28 kb)

Additional file 5: The Matlab® 2014b source codes for mathematical simulations. (ZIP 32 kb)

Additional file 6: The molecular profiles of CMS cancer cells, statistical significance analysis of reversion targets, and synergistic effect analysis of every two nodes inhibition. (XLSX 67 kb)

## Abbreviations

Akt: V-Akt murine thymoma viral oncogene homolog
APC: Adenomatous polyposis coli
ARF: Cyclin-dependent kinase inhibitor 2A
BRCA1: Breast cancer 1
CMS: Consensus Molecular Subtype
CNA: Copy number alteration
CPS: Cancer proximity score
E2F1: E2 family transcription factor 1
EGF: Epidermal growth factor
EMT: Epithelial-mesenchymal transition
GIANT: Genome-scale Integrated Analysis of gene Networks in Tissues
HPA: Human Protein Atlas
KEGG: Kyoto Encyclopedia of Genes and Genomes
KRAS: Kirsten-ras
MMP: Matrix metalloproteinase
p21: Cyclin-dependent kinase inhibitor 1A
PID: Pathway Interaction Database
PP2A: Protein phosphatase 2
PPARγ: Peroxisome proliferator-activated receptor γ
PPI: Protein-protein-interaction
PTEN: Tensin homolog
Rb: Retinoblastoma 1
TCGA: The Cancer and Geonome Atlas
TP53: Tumor protein p53
