# Supplementary material for: Quantitative evaluation and reversion analysis of the attractor landscapes of an intracellular regulatory network for colorectal cancer
Source: BMC Syst Biol. 2017 Apr 5;11:45. doi: 10.1186/s12918-017-0424-2 (PMC5382366; doi:10.1186/s12918-017-0424-2)
Supplement: Supplementary file 2 — Supporting texts and figures. (PDF 908 kb) [file 12918_2017_424_MOESM2_ESM.pdf]

Quantitative evaluation and reversion analysis of the attractor landscapes of an intracellular regulatory network for colorectal cancer

Yunseong Kim, Sea Choi, Dongkwan Shin, and Kwang-Hyun Cho*

Department of Bio and Brain Engineering, Korea Advanced Institute of Science and Technology (KAIST), Daejeon, Korea

Supporting Text and Figures

*Corresponding author. E-mail: ckh@kaist.ac.kr, Phone: +82-42-350-4325, Fax: +82-42-350-4310, Web: http://sbie.kaist.ac.kr/.

Text S1. Detailed descriptions of constructing colorectal cancer network

The main purpose of our study is to convert the cellular phenotype of a colorectal cancer cell into that of a normal cell. Hanahan *et al.* defined 10 functional phenotypes of cancer (“cancer hallmarks”) that indicate the transformation of normal cells to cancer cells, which include self-sufficiency in growth signals, evading apoptosis, insensitivity to anti-growth signals, sustained angiogenesis, limitless replicative potential, tissue invasion & metastasis, deregulating cellular energetics, avoiding immune destruction, genome instability and mutation, and tumor-promoting inflammation [1]. To investigate cancer reversion from a systems biological perspective, we have reconstructed an intracellular regulatory network model of a colorectal cancer cell by integrating all key molecules that can represent cancer hallmarks. As a result, we have included eight marker nodes closely related to uncontrolled proliferation, EMT, and stemness, which cover five cancer hallmarks: self-sufficiency in growth signals, evading apoptosis, insensitivity to anti-growth signals, tissue invasion & metastasis and genome instability and mutation. The other five cancer hallmarks such as sustained angiogenesis, limitless replicative potential, deregulating cellular energetics, avoiding immune destruction, and tumor-promoting inflammation were excluded since they are beyond the scope of our intracellular network model. Tissue invasion and metastasis, which are related to the EMT of colorectal cancer, can be represented by the expression level of E-cadherin which is a marker node of the EMT module in our network model [2]. Evading apoptosis, which is associated with the stemness of colorectal cancer, can be represented by the expression level of Snail and SLUG in the stemness module of our network [3]. Self-sufficiency in growth signals, which is related to the cell cycle of colorectal cancer, can be represented by the EGF signaling cascades and cyclin regulations which are marker nodes of the EGF module and cell cycle module of our network, respectively [4-6]. Insensitivity to anti-growth signals and genome instability and mutation, which are related to Wnt or DNA damage mediated apoptosis, can be represented by the Wnt signaling cascades and DNA damage recognition cascades which constitute the Wnt module and DNA damage module of our network, respectively [7, 8]. Taken together, we have considered three of the specific modules, EMT, cell cycle and stemness, in order to represent the functional phenotypes

of colorectal cancer with a minimum number of essential nodes. As shown in Fig. 1, there are six modules in our network which include the three modules that have a role as output marker control modules (Cell cycle, EMT and Stemness modules) whereas the other three modules take a role as input signal transduction modules (Wnt, EGF, and DNA damage modules). Each module has representative molecules, such as Wnt in the Wnt module and SLUG and Snail in the stemness module, and their interacting molecules are closely linked with each other and often form feedback loops in the network. Therefore, we can consider that the representative molecules and the interacting molecules are critical in determining the phenotype of each module.

Our network model of a colorectal cancer cell consists of six functional subnetworks in which Wnt, EGF, and DNA damage are related to input signals while cell cycle, EMT, and stemness are related to functional phenotypes of the network model in response to input signals. Wnt is an important input signal which determines the lineage of cellular phenotype such as EMT and proliferation in colorectal cancer [9]. The Wnt signal mediates the nuclear transportation of β-catenin and thereby promotes the expression of c-Myc by inhibiting the formation of DC (degradation complex) which is mainly composed of APC and GSK3β [10, 11]. In addition, EGF and DNA damage modules are most frequently altered subnetworks in colorectal cancer and are highly associated with two significant hallmarks of cancer, sustained proliferative signaling and resistance to cell death [12]. The EGF signaling cascades include KRAS, RAF, MEK, ERK and their inhibitory feedback regulators such as PP2A, ERK, Akt, and PAK [13]. ATM- or ARF-mediated DNA damage detection and p53-MDM2 feedback-mediated cell apoptosis induction are well-known and commonly used structures in DNA damage modules [14]. Cell cycle, EMT, and stemness modules are essential and most important modules in determining the phenotypic characteristics of colorectal cancer [2, 3]. The phase of cell cycle is determined by the amount of CyclinD or CyclinE, which are regulated by p21 and Rb, through E2F1 and Bcl-2 regulation [15]. Our network model focused on E-cadherin/MMP based cell adhesiveness regulation to describe the EMT function, regulated by NF-κB/IκB which is initiated by PTEN [16]. SLUG and Snail, critical marker genes for stemness of colorectal cancer, regulate BRCA1

which is closely related to DNA repair and drug resistance [3, 17]. In summary, we have integrated important subnetworks related to colorectal cancer and thereby established an essential intracellular regulatory network model of colorectal cancer by including critical marker genes that represent each subnetwork.

It is indeed true that SLUG and Snail are well-known regulators of EMT. On the other hand, they are also known as marker molecules highly associated with the stemness of colorectal cancer [3]. Recently, overexpression of Snail was shown to induce not only EMT but also a cancer stem cell-like phenotype in human colorectal cancer cells [18]. Moreover, Snail is known to be required for the expression of putative markers of stem cells, and can induce the expression of stemness-promoting genes, such as Nanog, KLF4, and TCF-4 [19]. Therefore, we can consider that SLUG and Snail might play critical roles in determining both EMT and stemness phenotypes in colorectal cancer. In our network model, the SLUG and Snail signals were transmitted to E-cadherin and MMP which are also known to be critical regulators of EMT in colorectal cancer. Hence, we chose E-cadherin and MMP as key marker genes of the EMT module to determine the functional phenotype of EMT, and assigned SLUG and Snail to the stemness module and set them as key marker molecules.

In this study, we aimed to construct a simple but essential regulatory network model of a colorectal cancer cell based on extensive literature survey and data-driven network inference. There are indeed several important regulators not included in our network model, such as TGFβ, SMAD4 or DCC. However, we have integrated the mutation and CNA profiles of these regulators when we construct the network model, so their regulatory effects were implicitly reflected in our network model. For instance, for a cell line with the genetic alteration of TGFβ, we fixed the expression levels of c-Myc and p21 as these are the target nodes of TGFβ [20, 21]. As a result, we could successfully reproduce the expression patterns and malignancy ranks of the cancer organoids, as shown in Fig. 3A [22]. Likewise, to reflect the effect of SMAD4, we controlled the expression levels of p21 and Snail [23, 24], which are regulated by SMAD4, and thereby we could successfully find out the cancer reversion phenomena (Fig. 3D). In summary, we tried

to construct a simple but essential regulatory network model with minimal nodes and to reflect implicitly the effects of some other missing regulators to the network wiring and regulatory logics. In this way, we have successfully recapitulated both the dynamics of the regulatory network of a colorectal cancer cell and previous experimental observations on cancer reversion phenomena.

Text S2. Boolean operator logic construction and weighted sum logic conversion

The states of biological nodes such as expression levels of genes or the activities of proteins can be represented by two quantitatively distinguishable states without any intermediate states. A Boolean network model is constructed solely with nodes that are either 'ON' or 'OFF' state in order to mimic the biological interactions effectively. Boolean network is beneficial for a network that contains well-defined structure of the nodes without having to know kinetic parameters. The three Boolean operators can regulate its nodes: AND, OR, and NOT. If a biological molecule acts inside a cell, it means that the molecule exists and be activated. If a biological molecule exists inside a cell, it means the molecule is frequently transcribed or stable. Thus, we can denote the state of a specific node in time step $t+1$ with the Boolean operator logic equation of input node states as below:

$$\boldsymbol{\delta_i^{t+1}} = (\boldsymbol{\delta_T^{t}} \ \vee \ \boldsymbol{\delta_S^{t}}) \ \wedge \ \boldsymbol{\delta_A^{t}}$$

Where $\boldsymbol{\delta_i^{t+1}}$ represents a molecular activity state of ith node at time step $t+1$, $\boldsymbol{\delta_T^{t}}$ represents input signals for transcriptional activation, $\boldsymbol{\delta_S^{t}}$ represents input signals for stabilization and $\boldsymbol{\delta_A^{t}}$ represents input signals for functional activation. The transcriptional activation is a biological interaction related with gene expression level changes such as transcription factor-target gene(TG-TF) interaction. The stabilization is a biological interactions related with molecular stability such as ubiquitination, degradation or polymer formation. The activation is a biological interactions related with molecular activity such as phosphorylation or methylation. As a result, we have constructed 34 Boolean operator logic equations based on the action mechanisms of each molecule in our network.

Although the Boolean operator logic is appropriate for reflecting biological action mechanisms to construct the update logic, it is hard to project the mutation and copy number variation data differently. Also, both defining the node state without any input node condition and adjusting the logic based on the experimental data are difficult in Boolean operator logic. The reason of these limitation is that there is no value where can adjust the node state quantitatively because the Boolean operator logic is a

qualitative equation. However, all of these problems can be solved when we use a weighted sum logic, a logic which defines both the basal node states and node relationships quantitatively as weights. Thus, not only projecting biological action mechanisms but also making easier to quantitatively adjust the relations, we have converted the Boolean operator logic equations into the weighted sum logic. In weighted sum logic, a network topology with N-nodes can be defined according to an N-by-N connectivity matrix, $\boldsymbol{M_C}$, where each matrix element $(\boldsymbol{M_R})_{ij}$ represents the connection from node j to node i. The tendency of each node's state without any incoming signal can be represented as a value in basal level of a column vector, $\boldsymbol{V_B}$. Gain of function mutation of KRAS and loss of function mutation of p53 and APC referred from The Cancer Genome Atlas (TCGA) databases, copy number variations (CNVs) amplification of BAX, SLUG, c-Myc, Rb, BRCA1, E2F1 and snail and loss of p53, Bcl-2 and APC referred from the big bang model of Sottoriva *et al.*, expression fold increase of E-cadherin, Bcl-2, Wnt referred from the Human Protein Atlas (HPA), were projected by adjusting the entity of $\boldsymbol{V_B}$. For each discrete time point t, the state of each node i, denoted $\boldsymbol{V_i^t}$, can be either 0 or 1. The input to each node is given by the weighted sum vector, $W_s = M_C \cdot V^t + V_B$. The next state $\boldsymbol{V_i^{t+1}}$ is then determined by the next input.

$$\boldsymbol{V_i^{t+1}} = \begin{cases} \mathbf{1} & (\boldsymbol{W_s})_i > \mathbf{0} \\ \boldsymbol{V_i^t} & (\boldsymbol{W_s})_i = \mathbf{0} \\ \mathbf{0} & (\boldsymbol{W_s})_i < \mathbf{0} \end{cases}$$

The basic algorithms of this conversion are (i) define the basal levels based on the prior biological knowledge, (ii) generate a standard truth table with the Boolean operator logic, (iii) generate every combination of weights in range of number of nodes, (iv) calculate the truth table for each combination of weights and compare the accordance with the standard truth table, (v) choose the weights combination with the best truth table accordance. The average accordance ratio is 97.74%. The logic conversion source codes and results are summarized in Additional file 2 and 4.

Figure S1

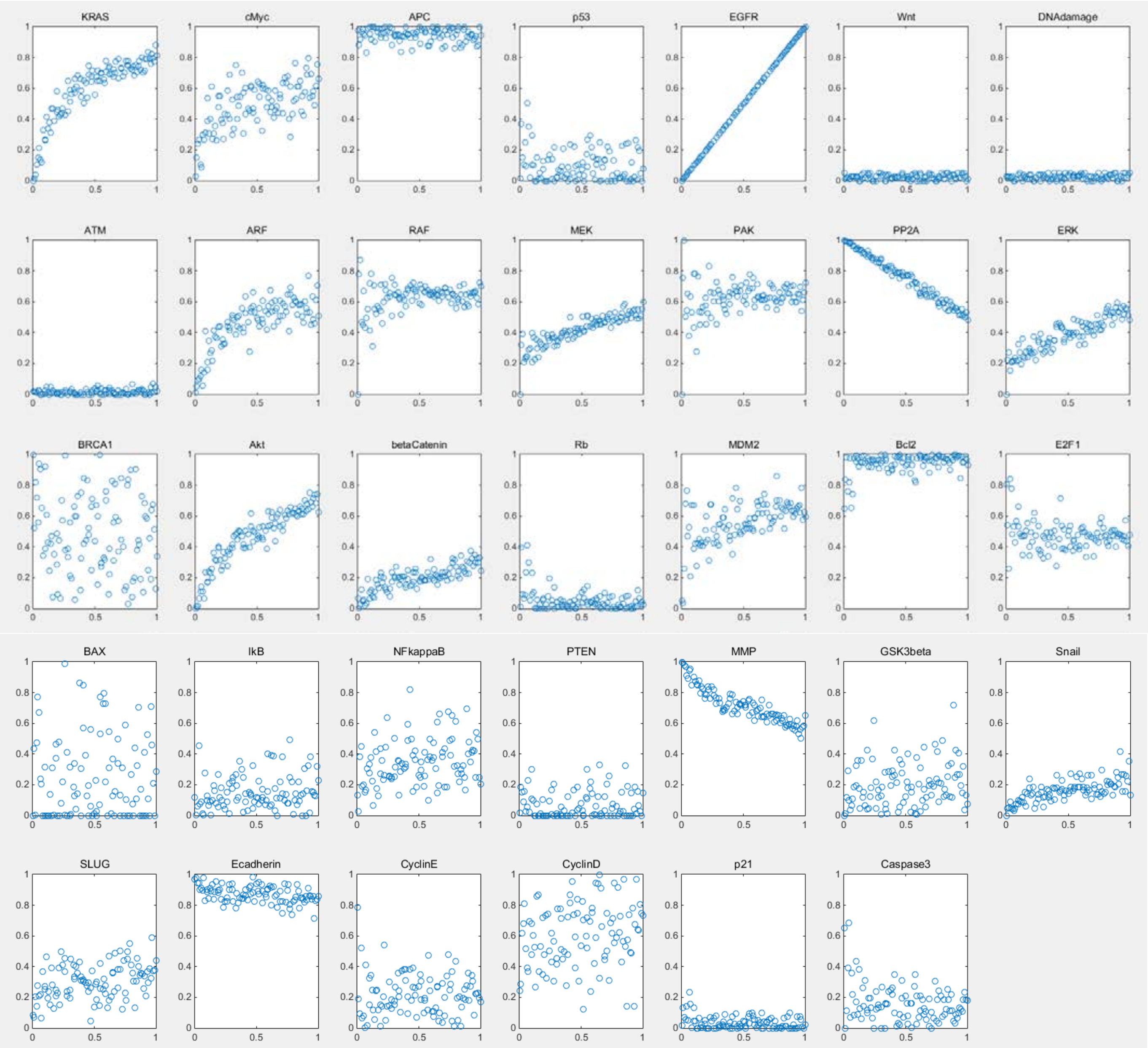

Figure S1. Biological and analytical validities of synchronous update logic. In our work, effects of the cancer reversion were observed only when we merged both gene regulatory network and protein-protein interaction network into one single network. Thus, we built a complex colorectal cancer network composed of both types of biological interactions intertwined each other. Because there is a variety of biological reaction times differs from one another in our combined network for cancer reversion, asynchronous update for our simulation analysis might be suggested. Previously, we also have used the deterministic asynchronous update logic, a weighted sum update logic which the connectivity matrix for each update

state changes as a subset of original connectivity matrix, based on the update probability of each links written in same entity of probability matrix. The result of node activation ratios based on input-output relationships of random asynchronous update logic in condition of gradual increase of EGFR activity is shown in this figure, which is similar pattern of synchronous update and implying the maintenance of the network dynamics. In addition, our analysis results have shown that more than 80% basin sizes were point attractors which indicate the normal-like scores of attractor landscapes from both of the update logics to be similar. We attempted to update every node in our network both synchronously and asynchronously, which in turn their point attractors were conserved with a slight change in reachability. Moreover, complex and irrelevant attractors were generated during the asynchronous updates which contained a vast number of network states that are not distinguishable within the degrees of cancerous states we created. These complex attractors have shown to be biologically unreliable since their states of the attractors were in mixture of both normal and cancer cell phenotypes. Thus, the synchronous update logic was not the only appropriate method for major attractors but biologically reliable for our network model. As a result, the synchronous update logic is finally chosen to be an update logic in our research.

Figure S2

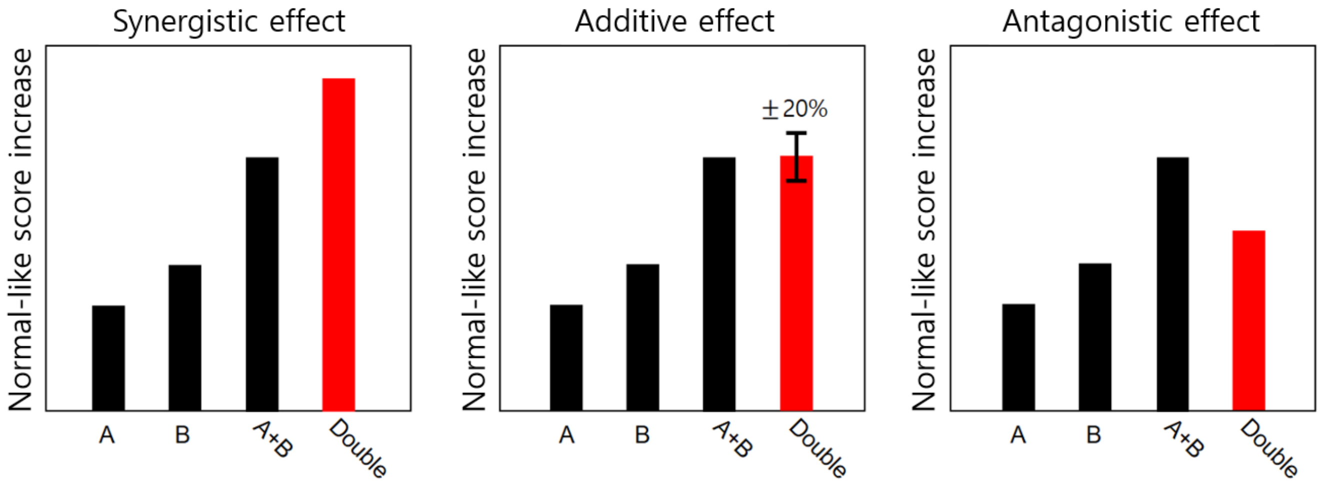

Figure S2. Quantitative classification of double node perturbation type. During the double node perturbation simulation, the associative effects of two perturbations should be able to quantitatively measure to reveal the interactive mechanism of nodes in the network. Thus, we have classified perturbation types into three: synergistic, additive and antagonistic. As shown in figure, we measure how much the normal-like score increases after performing a single- and a double-node perturbation with node A and B. If the increase of normal-like score during the double-node perturbation is larger, similar or smaller than the sum of the normal-like scores from the individually perturbed nodes A and B, then each incident can be classified as synergistic, additive or antagonistic, respectively. The range between increased normal-like scores has been calculated as 20% in average after performing double node perturbation in various pairs. As a result, we are able to separate the synergistic pairs from various combination of two nodes perturbations and analyze the specific mechanism of them which have lead us to reveal the functional motifs.

Figure S3

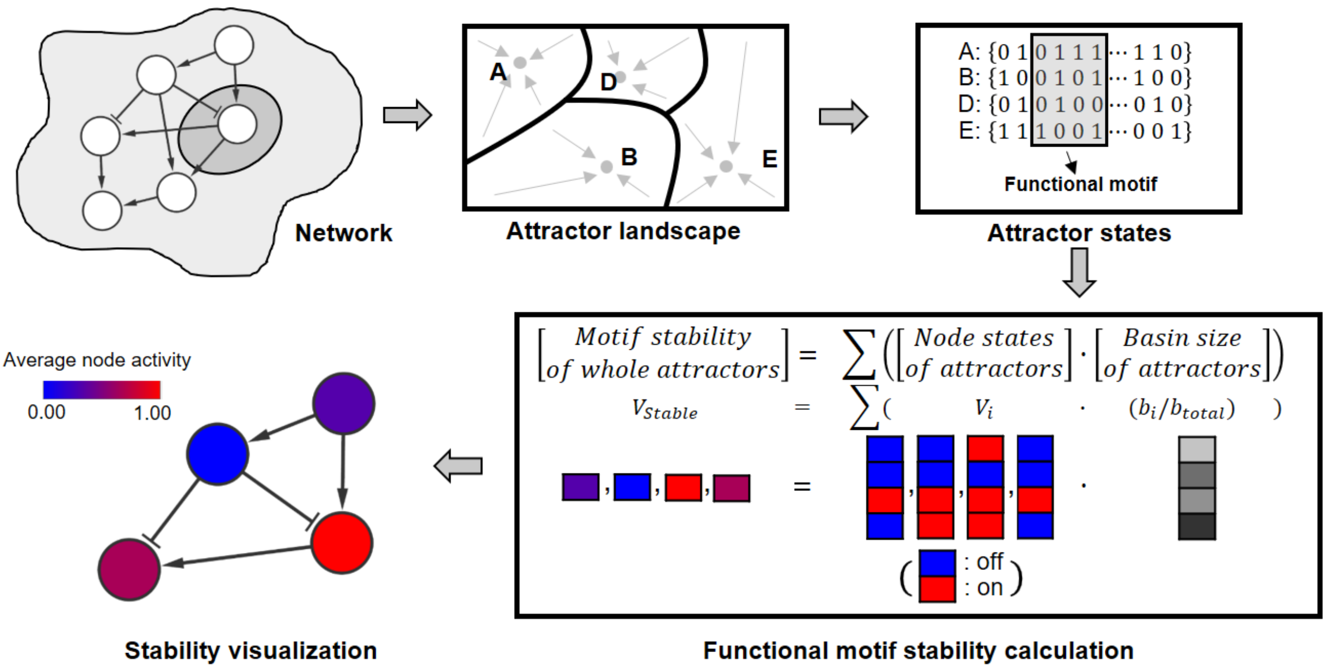

Figure S3. Motif stability analysis of colorectal cancer reversion functional motifs. The functional motifs which are revealed from motif analysis are basically curated from the condition of various node perturbations and mutations. Thus, even though there are some functional relationships in motifs, we need to proof the existence of the motifs in cancer cell. If the functional motif interacts more closely than other nodes, the node state combination of the motif will be pinned into a specific set regardless of the external signals or initial states. Thus, we tried to analyze the motif stability of functional motif in cancer network and check the effect of the cancer reversion target perturbation on that stability. The workflow of the motif stability analysis is shown in the figure. First, every attractor of initial states and its corresponding basin size is calculated. Second, the average activity of each node in the motif is calculated by adding the multiply of node state and basin size of each attractor. Third, visualize the stability of the network motif by coloring each node from blue to red.

Figure S4

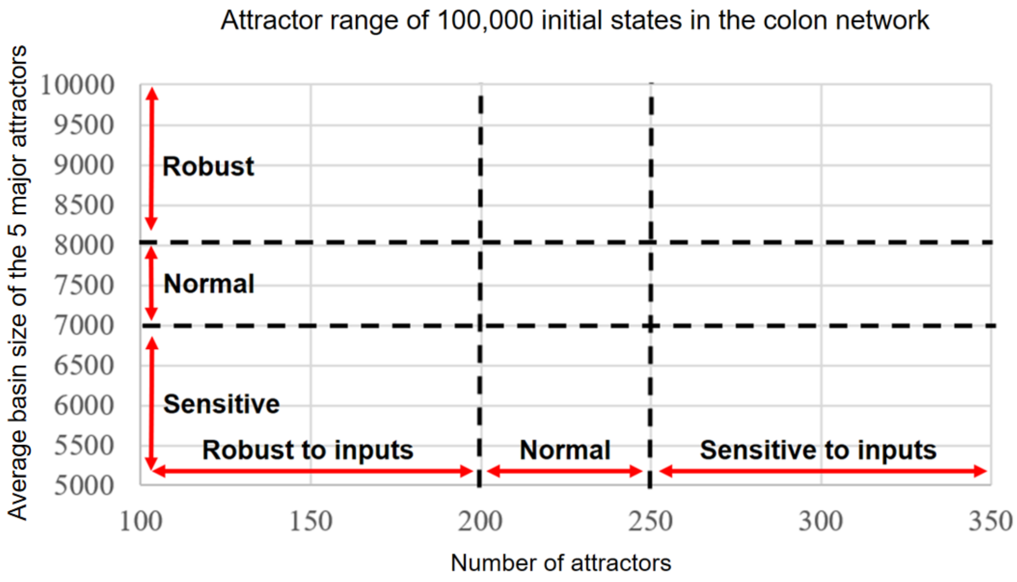

Figure S4. Robustness analysis of various sequences of mutations. To determine the robustness of the network against external perturbations, we examined the number of attractors and the average basin size of five major attractors. If the number of attractors is small or the average basin size of major attractors is large, external perturbation could hardly change the network state that stays in an attractor state, which makes the network robust to external perturbations. During the robustness analysis, the entire attractor landscapes of 100,000 initial states have been simulated while accumulating the node state fixation into either 0 or 1. In case of normal colon network, the number of attractors is around 220 while the average basin size of the five major attractors is around 7100. Thus, the normal colon network state is located in the middle dashed square area in the figure. During the mutation accumulation, the number of attractors and the average basin size of the five major attractors are changes. In this graphical presentation, if the network position is moved toward left or upper part of the coordinate, we can read that as an increase of the robustness of the network against the external signals. The simulations are repeated with the sequential mutations of the most commonly observed mutation sequence in colorectal cancer, the reverse sequence of the most commonly observed mutation sequence in colorectal cancer, the randomly selected mutation

sequences and determined each mutation type to decrease the normal-like score and the randomly selected mutation sequences. In case of the randomly selected mutation sequences and determined each mutation type to decrease the normal-like score, we have repeated the simulation 30 times and found the average pattern of the attractor changes.

Previous studies on the robustness analysis of Boolean networks mostly focused on the basin size of attraction as a robustness measure. For example, Li *et al.* considered the relative change in the basin size (B) of the largest attractor, $\Delta B/B$, as a measure of the network robustness when various network perturbations were applied [25]. They compared $\Delta B/B$ of the yeast cell-cycle network with that of a random network and demonstrated that the yeast cell-cycle network is more robust against the perturbation compared to the random network. Wang *et al.* computed the robustness of a network by calculating the basin size B and the trajectory overlap W based on the trajectory from every single state to its attractor [26]. They classified the edges of the yeast cell-cycle network and decomposed the network into spanning subnetworks and several smaller motifs, to select the nodes that are related to the attractor robustness against external network perturbation. Both studies used similar measures. First, they considered only the robustness of the largest attractor and did not consider the change of the whole attractor landscape. Second, the robustness of such a largest attractor was measured with the assumption that the change of state transition maps before and after perturbation can be fully traced. On the other hand, in our study, five major attractors corresponding to different phenotypes were considered with an attempt to evaluate the whole attractor landscape instead of focusing only on one major attractor. Note that, when we simulate the accumulation of multiple mutations during tumorigenesis, the distribution of the basin size of major attractors can vary quite a lot, so the largest attractor in one simulation step might not be the largest attractor in the next step. Moreover, as the state transition map can be dramatically changed at each simulation step over the accumulation of mutations, it might be difficult to calculate the robustness of attractors at every simulation step and to keep track of biologically meaningful attractors. In this respect, both $\Delta B/B$ and W might be inappropriate to evaluate our network model and can even result in undesirable outcomes. Since

the method of evaluating network robustness suggested in our study uses the average basin size of five major attractors rather than one single largest attractor, our method can be better in measuring the robustness of the whole network. Note also that we used both basin size and number of attractors to measure the network robustness, which enables to determine the ranking of network robustness at each tumorigenesis step. Using this concept of network robustness, we were able to trace the network robustness during tumorigenesis and could finally use the information to find out a novel strategy for cancer reversion.

Figure S5

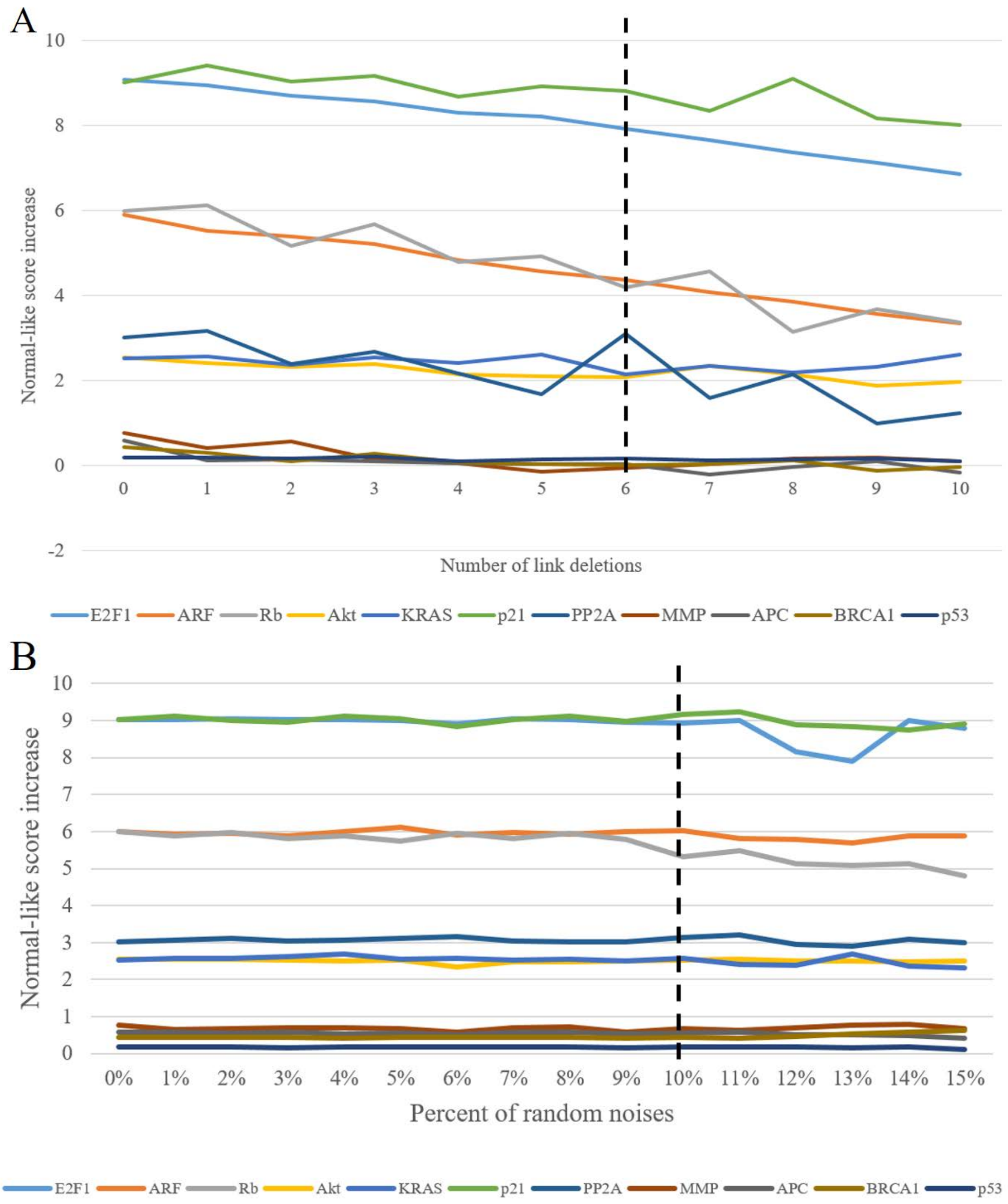

Figure S5. Robustness analysis of the network model for the random link deletion or the white noise perturbation. We have further examined the robustness of the structure and dynamics of our network model by investigating the increase of normal-like score with respect to controlling 11 suggested cancer

reversion targets along with random deletion of network links. To examine the structural robustness of our network model, we have randomly deleted up to 10 links of our network model. We have simulated all possible combinations of link deletion from single to three links and have randomly sampled 100,000 combinations for four or more link deletion due to computational limitation. After all, we have calculated the average increase of normal-like score for every case. We found that our network model is reasonably robust with respect to the increase of normal-like score for about six random link deletion (see Fig. S5A), indicating that our results remain robust even if the network structure is changed in that range. In addition, to check the dynamical robustness of our network model, we have added random noise from 0 to 15% of each element value of the connectivity matrix and the basal level column vector. After each simulation, we have calculated the average increase of normal-like score for all different levels of random noise. We found that our network model again is reasonably robust against up to 10% noise level (see Fig. S5B), indicating that the dynamical property of our network model is robust against such perturbations.

Figure S6

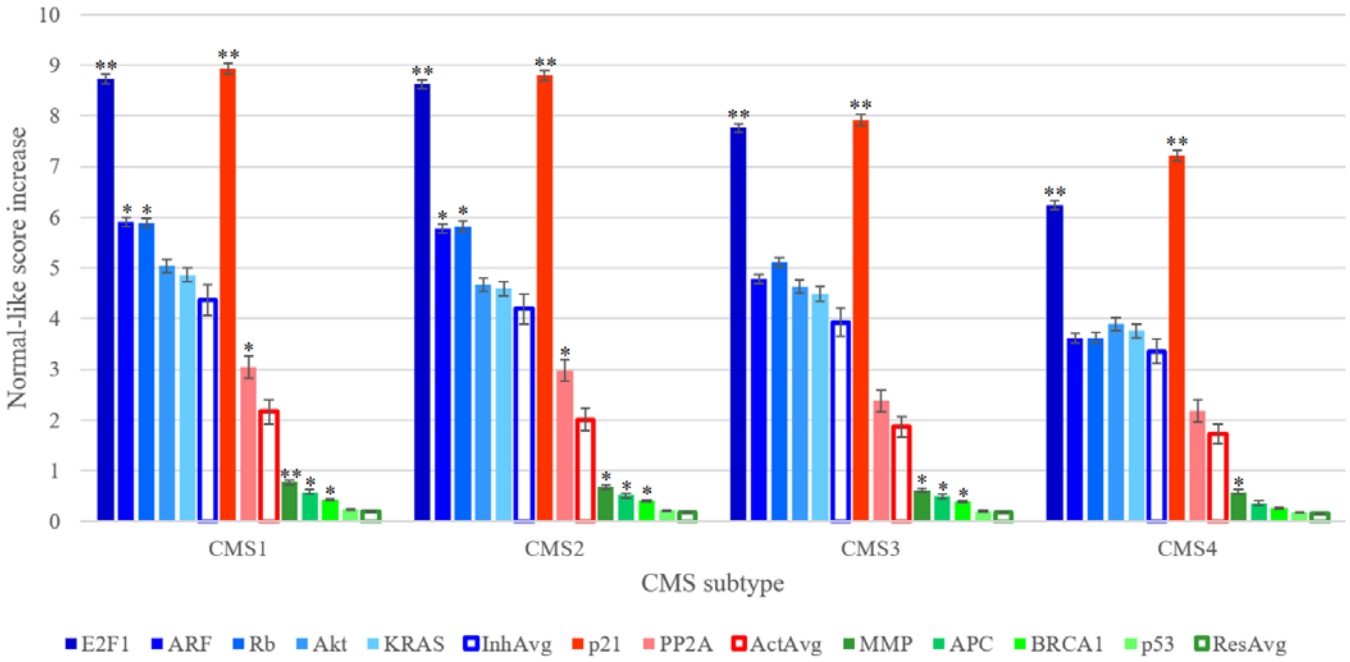

Figure S6. The statistical significance of 11 suggested cancer reversion targets. We tested the statistical significance of cancer reversion by simulating the increase of normal-like score for three different types of perturbations (activation, inhibition, and restoration) on 34 nodes in 49 cell lines (the wild type and 4 CMSs of 12 cell lines) [27]. As a result, the cancer reversion for the perturbation of the suggested 11 cancer reversion targets is found to be statistically significant (Fig. S6), which indicates that the increase of the normal-like score is large enough to confirm successful cancer reversion by controlling the suggested targets. The one or two stars on top of each bar of the graph represent the p-value below 0.05 or 0.001 respectively. Blue, red and green bar in the figure represent three different types of perturbations, activation, inhibition, and restoration, respectively. The increase of the normal-like score for most of the reversion targets was found to be statistically significant compared to the average increase of the normal-like score when we perturb non-reversion targets as indicated by an empty bar with solid line according to each perturbation type.

Figure S7

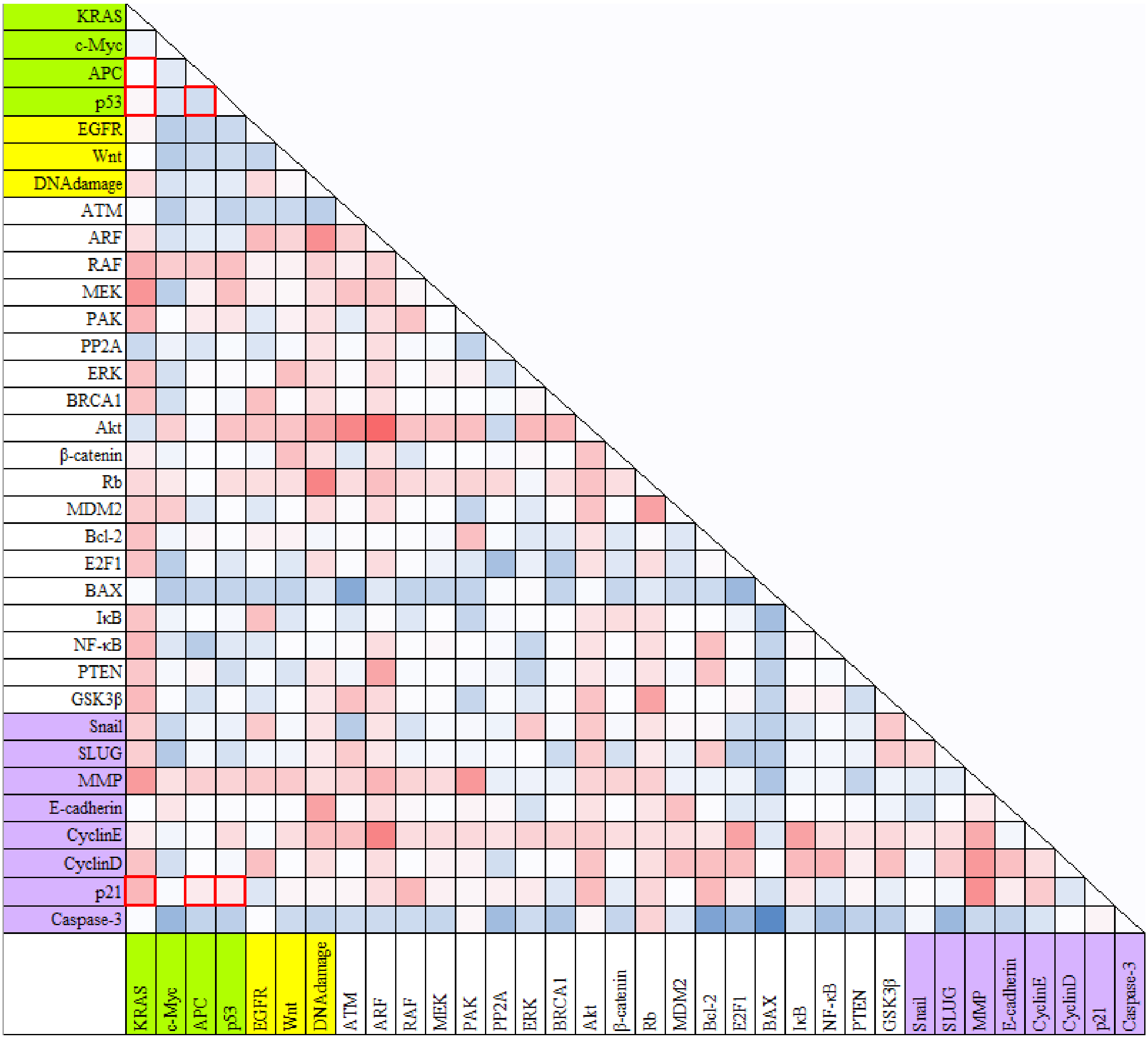

Figure S7. Double nodes perturbation effects for every pairs of 34 nodes. We have simulated the synergistic effects of cancer reversion for every pair of 34 nodes in our network including APC, p53, KRAS and p21(SMAD4), which were known to induce colorectal cancer when sequentially mutated. As a result, no significant synergistic effect was observed among these pairs, as shown in Fig. R4. Although Bax and Caspase-3 are likely to be synergistic for every pair, they are not synergistic in terms of cancer reversion since they continuously induce apoptosis. Except for them, almost every synergistic pairs are included in the stable motifs shown in Fig. 5A. The pairs among APC, P53, KRAS and SMAD4 are not significantly

synergistic, and even some of the pairs, such as three pairs with p21, are antagonistic. As we mentioned in the EGF signal transmission motif in Fig. 4C, the synergy of cancer reversion can emerge when multiple nodes of a single signaling pathway were perturbed simultaneously. Thus, adjacent nodes can have a higher chance to cause such a synergistic effect than distant nodes. However, we found that four of the frequently mutated nodes in colorectal cancer are located in different modules and are relatively far apart in our network model, which might be one of the reasons why no significant synergistic effect was observed among them. The blue color indicates a synergistic pair while the red color indicates an antagonistic pair. The pairs among APC, p53, KRAS, and p21 (SMAD4) were denoted by red squares. The darker the color, the more synergistic or antagonistic the pair is.

References

1. Hanahan D, Weinberg RA: **Hallmarks of cancer: the next generation**. *cell* 2011, **144**(5):646-674.
2. Sadanandam A, Lyssiotis CA, Homicsko K, Collisson EA, Gibb WJ, Wullschleger S, Ostos LCG, Lannon WA, Grotzinger C, Del Rio M: **A colorectal cancer classification system that associates cellular phenotype and responses to therapy**. *Nature medicine* 2013, **19**(5):619-625.
3. Hwang WL, Yang MH, Tsai ML, Lan HY, Su SH, Chang SC, Teng HW, Yang SH, Lan YT, Chiou SH: **SNAIL regulates interleukin-8 expression, stem cell–like activity, and tumorigenicity of human colorectal carcinoma cells**. *Gastroenterology* 2011, **141**(1):279-291. e275.
4. Arrington AK, Heinrich EL, Lee W, Duldulao M, Patel S, Sanchez J, Garcia-Aguilar J, Kim J: **Prognostic and predictive roles of KRAS mutation in colorectal cancer**. *International journal of molecular sciences* 2012, **13**(10):12153-12168.
5. Mendoza MC, Er EE, Blenis J: **The Ras-ERK and PI3K-mTOR pathways: cross-talk and compensation**. *Trends in biochemical sciences* 2011, **36**(6):320-328.
6. Fauré A, Naldi A, Chaouiya C, Thieffry D: **Dynamical analysis of a generic Boolean model for the control of the mammalian cell cycle**. *Bioinformatics* 2006, **22**(14):e124-e131.
7. Najdi R, Holcombe RF, Waterman ML: **Wnt signaling and colon carcinogenesis: beyond APC**. *Journal of carcinogenesis* 2011, **10**(1):5.
8. Halazonetis TD, Gorgoulis VG, Bartek J: **An oncogene-induced DNA damage model for cancer development**. *science* 2008, **319**(5868):1352-1355.
9. Shin S-Y, Rath O, Zebisch A, Choo S-M, Kolch W, Cho K-H: **Functional roles of multiple feedback loops in extracellular signal-regulated kinase and Wnt signaling pathways that regulate epithelial-mesenchymal transition**. *Cancer research* 2010, **70**(17):6715-6724.
10. Song J-H, Huels DJ, Ridgway RA, Sansom OJ, Kholodenko BN, Kolch W, Cho K-H: **The APC network regulates the removal of mutated cells from colonic crypts**. *Cell reports* 2014, **7**(1):94-103.
11. He T-C, Sparks AB, Rago C, Hermeking H, Zawel L, Da Costa LT, Morin PJ, Vogelstein B, Kinzler KW: **Identification of c-MYC as a target of the APC pathway**. *Science* 1998, **281**(5382):1509-1512.
12. Kreeger PK, Lauffenburger DA: **Cancer systems biology: a network modeling perspective**. *Carcinogenesis* 2010, **31**(1):2-8.
13. Won J-K, Yang HW, Shin S-Y, Lee JH, Do Heo W, Cho K-H: **The crossregulation between ERK and PI3K signaling pathways determines the tumoricidal efficacy of MEK inhibitor**. *Journal*

of molecular cell biology 2012, **4**(3):153-163.

14. Choi M, Shi J, Jung SH, Chen X, Cho K-H: **Attractor landscape analysis reveals feedback loops in the p53 network that control the cellular response to DNA damage**. *Sci Signal* 2012, **5**(251):83.
15. Lo H, Hung M: **Nuclear EGFR signalling network in cancers: linking EGFR pathway to cell cycle progression, nitric oxide pathway and patient survival**. *British journal of cancer* 2006, **94**(2):184-188.
16. Chen B-S, Wu C-C: **Systems biology as an integrated platform for bioinformatics, systems synthetic biology, and systems metabolic engineering**. *Cells* 2013, **2**(4):635-688.
17. Niell BL, Rennert G, Bonner JD, Almog R, Tomsho LP, Gruber SB: **BRCA1 and BRCA2 founder mutations and the risk of colorectal cancer**. *Journal of the National Cancer Institute* 2004, **96**(1):15-21.
18. Fan F, Samuel S, Evans KW, Lu J, Xia L, Zhou Y, Sceusi E, Tozzi F, Ye XC, Mani SA: **Overexpression of Snail induces epithelial–mesenchymal transition and a cancer stem cell–like phenotype in human colorectal cancer cells**. *Cancer medicine* 2012, **1**(1):5-16.
19. Kurrey NK, Jalgaonkar SP, Joglekar AV, Ghanate AD, Chaskar PD, Doiphode RY, Bapat SA: **Snail and slug mediate radioresistance and chemoresistance by antagonizing p53-mediated apoptosis and acquiring a stem-like phenotype in ovarian cancer cells**. *Stem cells* 2009, **27**(9):2059-2068.
20. Dews M, Tan GS, Hultine S, Raman P, Choi J, Duperret EK, Lawler J, Bass A, Thomas-Tikhonenko A: **Masking epistasis between MYC and TGF-β pathways in antiangiogenesis-mediated colon cancer suppression**. *Journal of the National Cancer Institute* 2014, **106**(4):dju043.
21. Bauer J, Sporn JC, Cabral J, Gomez J, Jung B: **Effects of activin and TGFβ on p21 in colon cancer**. *PloS one* 2012, **7**(6):e39381.
22. Drost J, Van Jaarsveld RH, Ponsioen B, Zimberlin C, Van Boxtel R, Buijs A, Sachs N, Overmeer RM, Offerhaus GJ, Begthel H: **Sequential cancer mutations in cultured human intestinal stem cells**. *Nature* 2015, **521**(7550):43-47.
23. Matsuzaki K, Kitano C, Murata M, Sekimoto G, Yoshida K, Uemura Y, Seki T, Taketani S, Fujisawa J-i, Okazaki K: **Smad2 and Smad3 phosphorylated at both linker and COOH-terminal regions transmit malignant TGF-β signal in later stages of human colorectal cancer**. *Cancer research* 2009, **69**(13):5321-5330.
24. Reinacher-Schick A, Baldus SE, Romdhana B, Landsberg S, Zapatka M, Mönig SP, Hölscher AH, Dienes HP, Schmiegel W, Schwarte-Waldhoff I: **Loss of Smad4 correlates with loss of the invasion suppressor E-cadherin in advanced colorectal carcinomas**. *The Journal of pathology*

2004, **202**(4):412-420.

25. Li F, Long T, Lu Y, Ouyang Q, Tang C: **The yeast cell-cycle network is robustly designed**. *Proceedings of the National Academy of Sciences of the United States of America* 2004, **101**(14):4781-4786.
26. Wang G, Du C, Chen H, Simha R, Rong Y, Xiao Y, Zeng C: **Process-based network decomposition reveals backbone motif structure**. *Proceedings of the National Academy of Sciences* 2010, **107**(23):10478-10483.
27. Guinney J, Dienstmann R, Wang X, De Reyniès A, Schlicker A, Soneson C, Marisa L, Roepman P, Nyamundanda G, Angelino P: **The consensus molecular subtypes of colorectal cancer**. *Nature medicine* 2015.
